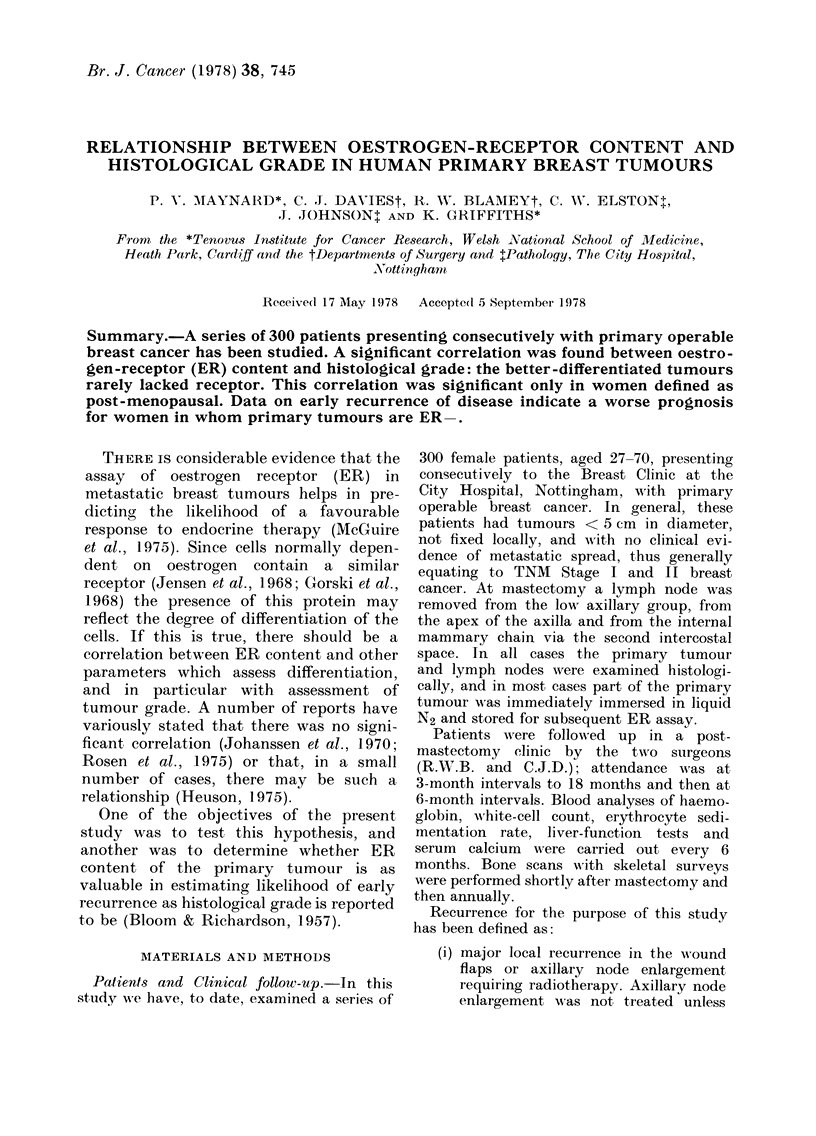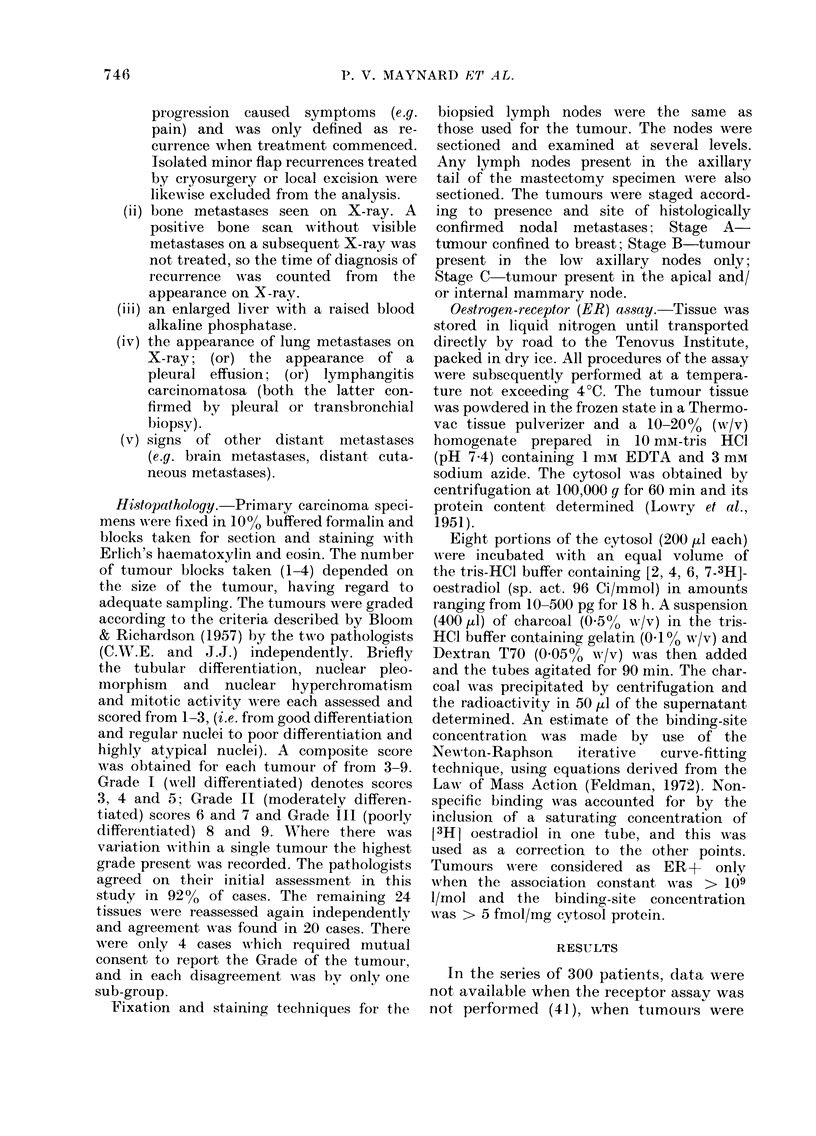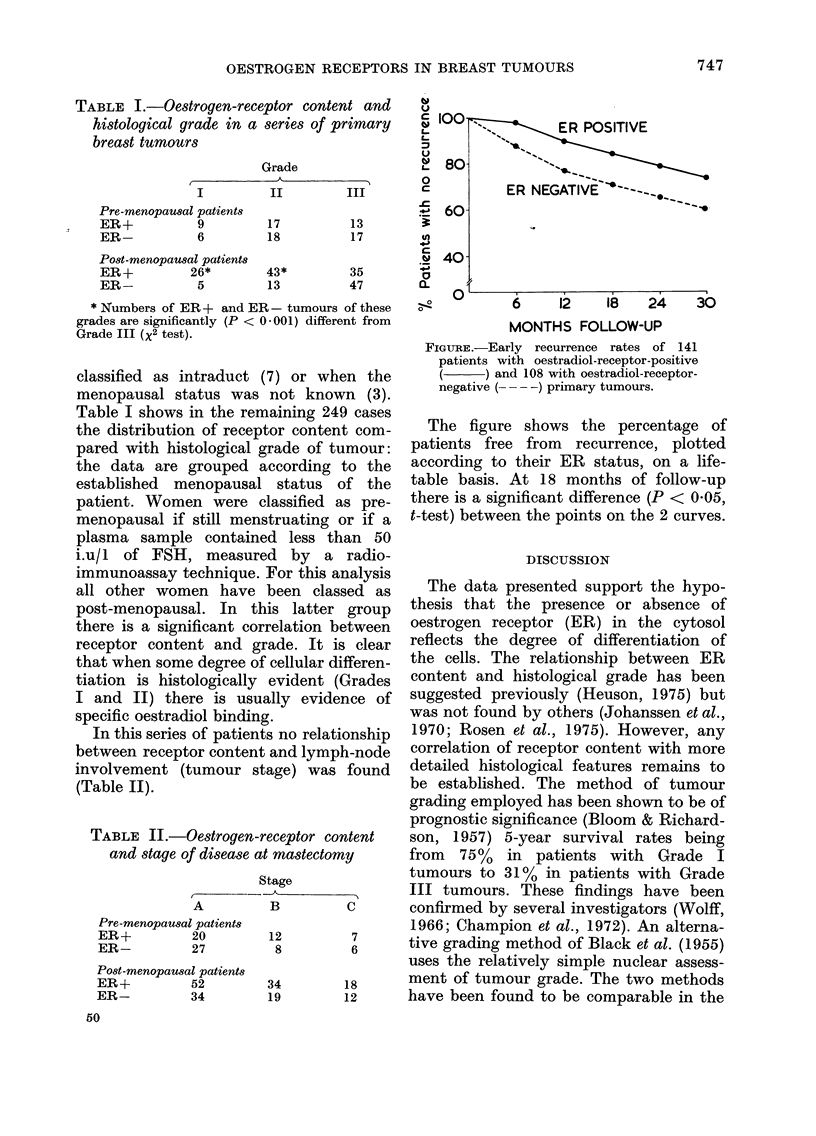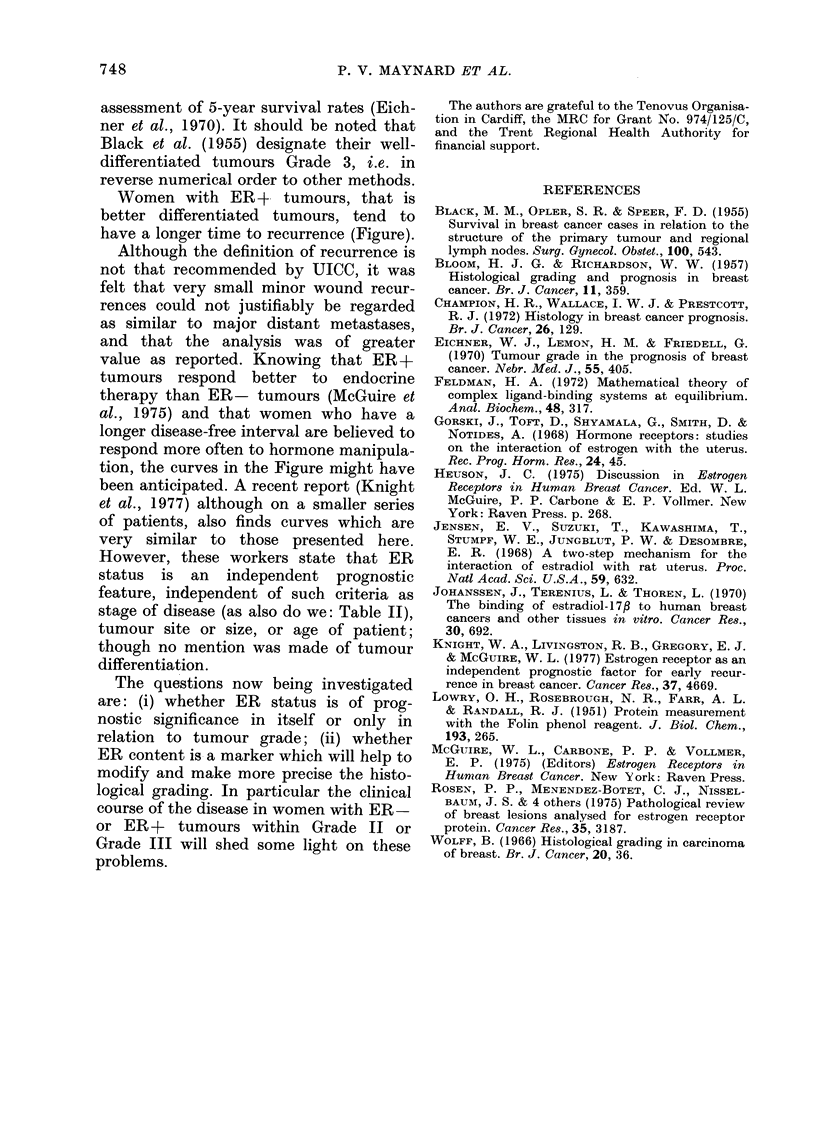# Relationship between oestrogen-receptor content and histological grade in human primary breast tumours.

**DOI:** 10.1038/bjc.1978.282

**Published:** 1978-12

**Authors:** P. V. Maynard, C. J. Davies, R. W. Blamey, C. W. Elston, J. Johnson, K. Griffiths

## Abstract

A series of 300 patients presenting consecutively with primary operable breast cancer has been studied. A significant correlation was found between oestrogen-receptor (ER) content and histological grade: the better-differentiated tumours rarely lacked receptor. This correlation was significant only in women defined as post-menopausal. Data on early recurrence of disease indicate a worse prognosis for women in whom primary tumours are ER-.


*Br. J. Cancer* (1978) **38**, 745

### RELATIONSHIP BETWEEN OESTROGEN-RECEPTOR CONTENT AND HISTOLOGICAL GRADE IN HUMAN PRIMARY BREAST TUMOURS

P. V. MAYNARD*, C. J. DAVIES†, R. W. BLAMEY†, C. W. ELSTON‡, J. JOHNSON‡ AND K. GRIFFITHS*

*From the *Tenovus Institute for Cancer Research, Welsh National School of Medicine, Heath Park, Cardiff and the †Departments of Surgery and ‡Pathology, The City Hospital, Nottingham*

Received 17 May 1978 Accepted 5 September 1978

**Summary.—A series of 300 patients presenting consecutively with primary operable breast cancer has been studied. A significant correlation was found between oestrogen-receptor (ER) content and histological grade: the better-differentiated tumours rarely lacked receptor. This correlation was significant only in women defined as post-menopausal. Data on early recurrence of disease indicate a worse prognosis for women in whom primary tumours are ER−.**

THERE IS considerable evidence that the assay of oestrogen receptor (ER) in metastatic breast tumours helps in predicting the likelihood of a favourable response to endocrine therapy (McGuire *et al.*, 1975). Since cells normally dependent on oestrogen contain a similar receptor (Jensen *et al.*, 1968; Gorski *et al.*, 1968) the presence of this protein may reflect the degree of differentiation of the cells. If this is true, there should be a correlation between ER content and other parameters which assess differentiation, and in particular with assessment of tumour grade. A number of reports have variously stated that there was no significant correlation (Johanssen *et al.*, 1970; Rosen *et al.*, 1975) or that, in a small number of cases, there may be such a relationship (Heuson, 1975).

One of the objectives of the present study was to test this hypothesis, and another was to determine whether ER content of the primary tumour is as valuable in estimating likelihood of early recurrence as histological grade is reported to be (Bloom & Richardson, 1957).

### MATERIALS AND METHODS

*Patients and Clinical follow-up.*—In this study we have, to date, examined a series of 300 female patients, aged 27–70, presenting consecutively to the Breast Clinic at the City Hospital, Nottingham, with primary operable breast cancer. In general, these patients had tumours $< 5$ cm in diameter, not fixed locally, and with no clinical evidence of metastatic spread, thus generally equating to TNM Stage I and II breast cancer. At mastectomy a lymph node was removed from the low axillary group, from the apex of the axilla and from the internal mammary chain via the second intercostal space. In all cases the primary tumour and lymph nodes were examined histologically, and in most cases part of the primary tumour was immediately immersed in liquid $N_2$ and stored for subsequent ER assay.

Patients were followed up in a post-mastectomy clinic by the two surgeons (R.W.B. and C.J.D.); attendance was at 3-month intervals to 18 months and then at 6-month intervals. Blood analyses of haemoglobin, white-cell count, erythrocyte sedimentation rate, liver-function tests and serum calcium were carried out every 6 months. Bone scans with skeletal surveys were performed shortly after mastectomy and then annually.

Recurrence for the purpose of this study has been defined as:

(i) major local recurrence in the wound flaps or axillary node enlargement requiring radiotherapy. Axillary node enlargement was not treated unless progression caused symptoms (*e.g.* pain) and was only defined as recurrence when treatment commenced. Isolated minor flap recurrences treated by cryosurgery or local excision were likewise excluded from the analysis.

(ii) bone metastases seen on X-ray. A positive bone scan without visible metastases on a subsequent X-ray was not treated, so the time of diagnosis of recurrence was counted from the appearance on X-ray.

(iii) an enlarged liver with a raised blood alkaline phosphatase.

(iv) the appearance of lung metastases on X-ray; (or) the appearance of a pleural effusion; (or) lymphangitis carcinomatosa (both the latter confirmed by pleural or transbronchial biopsy).

(v) signs of other distant metastases (*e.g.* brain metastases, distant cutaneous metastases).

*Histopathology.*—Primary carcinoma specimens were fixed in 10% buffered formalin and blocks taken for section and staining with Erlich's haematoxylin and eosin. The number of tumour blocks taken (1–4) depended on the size of the tumour, having regard to adequate sampling. The tumours were graded according to the criteria described by Bloom & Richardson (1957) by the two pathologists (C.W.E. and J.J.) independently. Briefly the tubular differentiation, nuclear pleomorphism and nuclear hyperchromatism and mitotic activity were each assessed and scored from 1–3, (*i.e.* from good differentiation and regular nuclei to poor differentiation and highly atypical nuclei). A composite score was obtained for each tumour of from 3–9. Grade I (well differentiated) denotes scores 3, 4 and 5; Grade II (moderately differentiated) scores 6 and 7 and Grade III (poorly differentiated) 8 and 9. Where there was variation within a single tumour the highest grade present was recorded. The pathologists agreed on their initial assessment in this study in 92% of cases. The remaining 24 tissues were reassessed again independently and agreement was found in 20 cases. There were only 4 cases which required mutual consent to report the Grade of the tumour, and in each disagreement was by only one sub-group.

Fixation and staining techniques for the biopsied lymph nodes were the same as those used for the tumour. The nodes were sectioned and examined at several levels. Any lymph nodes present in the axillary tail of the mastectomy specimen were also sectioned. The tumours were staged according to presence and site of histologically confirmed nodal metastases; Stage A—tumour confined to breast; Stage B—tumour present in the low axillary nodes only; Stage C—tumour present in the apical and/or internal mammary node.

*Oestrogen-receptor* (*ER*) *assay.*—Tissue was stored in liquid nitrogen until transported directly by road to the Tenovus Institute, packed in dry ice. All procedures of the assay were subsequently performed at a temperature not exceeding 4°C. The tumour tissue was powdered in the frozen state in a Thermovac tissue pulverizer and a 10–20% (w/v) homogenate prepared in 10 mM-tris HCl (pH 7·4) containing 1 mM EDTA and 3 mM sodium azide. The cytosol was obtained by centrifugation at 100,000 *g* for 60 min and its protein content determined (Lowry *et al.*, 1951).

Eight portions of the cytosol (200 $\mu$l each) were incubated with an equal volume of the tris-HCl buffer containing [2, 4, 6, 7-$^3$H]-oestradiol (sp. act. 96 Ci/mmol) in amounts ranging from 10–500 pg for 18 h. A suspension (400 $\mu$l) of charcoal (0·5% w/v) in the tris-HCl buffer containing gelatin (0·1% w/v) and Dextran T70 (0·05% w/v) was then added and the tubes agitated for 90 min. The charcoal was precipitated by centrifugation and the radioactivity in 50 $\mu$l of the supernatant determined. An estimate of the binding-site concentration was made by use of the Newton-Raphson iterative curve-fitting technique, using equations derived from the Law of Mass Action (Feldman, 1972). Non-specific binding was accounted for by the inclusion of a saturating concentration of [$^3$H] oestradiol in one tube, and this was used as a correction to the other points. Tumours were considered as ER+ only when the association constant was $> 10^9$ l/mol and the binding-site concentration was $>$ 5 fmol/mg cytosol protein.

### RESULTS

In the series of 300 patients, data were not available when the receptor assay was not performed (41), when tumours were

TABLE I.—*Oestrogen-receptor content and histological grade in a series of primary breast tumours*

| | Grade | | |
|---|---|---|---|
| | I | II | III |
| *Pre-menopausal patients* | | | |
| ER+ | 9 | 17 | 13 |
| ER− | 6 | 18 | 17 |
| *Post-menopausal patients* | | | |
| ER+ | 26* | 43* | 35 |
| ER− | 5 | 13 | 47 |

* Numbers of ER+ and ER− tumours of these grades are significantly ($P < 0{\cdot}001$) different from Grade III ($\chi^2$ test).

FIGURE.—Early recurrence rates of 141 patients with oestradiol-receptor-positive (———) and 108 with oestradiol-receptor-negative (– – – –) primary tumours.

classified as intraduct (7) or when the menopausal status was not known (3). Table I shows in the remaining 249 cases the distribution of receptor content compared with histological grade of tumour: the data are grouped according to the established menopausal status of the patient. Women were classified as pre-menopausal if still menstruating or if a plasma sample contained less than 50 i.u/l of FSH, measured by a radio-immunoassay technique. For this analysis all other women have been classed as post-menopausal. In this latter group there is a significant correlation between receptor content and grade. It is clear that when some degree of cellular differentiation is histologically evident (Grades I and II) there is usually evidence of specific oestradiol binding.

In this series of patients no relationship between receptor content and lymph-node involvement (tumour stage) was found (Table II).

TABLE II.—*Oestrogen-receptor content and stage of disease at mastectomy*

| | Stage | | |
|---|---|---|---|
| | A | B | C |
| *Pre-menopausal patients* | | | |
| ER+ | 20 | 12 | 7 |
| ER− | 27 | 8 | 6 |
| *Post-menopausal patients* | | | |
| ER+ | 52 | 34 | 18 |
| ER− | 34 | 19 | 12 |

The figure shows the percentage of patients free from recurrence, plotted according to their ER status, on a life-table basis. At 18 months of follow-up there is a significant difference ($P < 0{\cdot}05$, *t*-test) between the points on the 2 curves.

DISCUSSION

The data presented support the hypothesis that the presence or absence of oestrogen receptor (ER) in the cytosol reflects the degree of differentiation of the cells. The relationship between ER content and histological grade has been suggested previously (Heuson, 1975) but was not found by others (Johanssen *et al.*, 1970; Rosen *et al.*, 1975). However, any correlation of receptor content with more detailed histological features remains to be established. The method of tumour grading employed has been shown to be of prognostic significance (Bloom & Richardson, 1957) 5-year survival rates being from 75% in patients with Grade I tumours to 31% in patients with Grade III tumours. These findings have been confirmed by several investigators (Wolff, 1966; Champion *et al.*, 1972). An alternative grading method of Black *et al.* (1955) uses the relatively simple nuclear assessment of tumour grade. The two methods have been found to be comparable in the assessment of 5-year survival rates (Eichner *et al.*, 1970). It should be noted that Black *et al.* (1955) designate their well-differentiated tumours Grade 3, *i.e.* in reverse numerical order to other methods.

Women with ER+ tumours, that is better differentiated tumours, tend to have a longer time to recurrence (Figure).

Although the definition of recurrence is not that recommended by UICC, it was felt that very small minor wound recurrences could not justifiably be regarded as similar to major distant metastases, and that the analysis was of greater value as reported. Knowing that ER+ tumours respond better to endocrine therapy than ER− tumours (McGuire *et al.*, 1975) and that women who have a longer disease-free interval are believed to respond more often to hormone manipulation, the curves in the Figure might have been anticipated. A recent report (Knight *et al.*, 1977) although on a smaller series of patients, also finds curves which are very similar to those presented here. However, these workers state that ER status is an independent prognostic feature, independent of such criteria as stage of disease (as also do we: Table II), tumour site or size, or age of patient; though no mention was made of tumour differentiation.

The questions now being investigated are: (i) whether ER status is of prognostic significance in itself or only in relation to tumour grade; (ii) whether ER content is a marker which will help to modify and make more precise the histological grading. In particular the clinical course of the disease in women with ER− or ER+ tumours within Grade II or Grade III will shed some light on these problems.

The authors are grateful to the Tenovus Organisation in Cardiff, the MRC for Grant No. 974/125/C, and the Trent Regional Health Authority for financial support.

### REFERENCES

Black, M. M., Opler, S. R. & Speer, F. D. (1955) Survival in breast cancer cases in relation to the structure of the primary tumour and regional lymph nodes. *Surg. Gynecol. Obstet.*, **100**, 543.

Bloom, H. J. G. & Richardson, W. W. (1957) Histological grading and prognosis in breast cancer. *Br. J. Cancer*, **11**, 359.

Champion, H. R., Wallace, I. W. J. & Prestcott, R. J. (1972) Histology in breast cancer prognosis. *Br. J. Cancer*, **26**, 129.

Eichner, W. J., Lemon, H. M. & Friedell, G. (1970) Tumour grade in the prognosis of breast cancer. *Nebr. Med. J.*, **55**, 405.

Feldman, H. A. (1972) Mathematical theory of complex ligand-binding systems at equilibrium. *Anal. Biochem.*, **48**, 317.

Gorski, J., Toft, D., Shyamala, G., Smith, D. & Notides, A. (1968) Hormone receptors: studies on the interaction of estrogen with the uterus. *Rec. Prog. Horm. Res.*, **24**, 45.

Heuson, J. C. (1975) Discussion in *Estrogen Receptors in Human Breast Cancer*. Ed. W. L. McGuire, P. P. Carbone & E. P. Vollmer. New York: Raven Press. p. 268.

Jensen, E. V., Suzuki, T., Kawashima, T., Stumpf, W. E., Jungblut, P. W. & Desombre, E. R. (1968) A two-step mechanism for the interaction of estradiol with rat uterus. *Proc. Natl Acad. Sci. U.S.A.*, **59**, 632.

Johanssen, J., Terenius, L. & Thoren, L. (1970) The binding of estradiol-17$\beta$ to human breast cancers and other tissues *in vitro*. *Cancer Res.*, **30**, 692.

Knight, W. A., Livingston, R. B., Gregory, E. J. & McGuire, W. L. (1977) Estrogen receptor as an independent prognostic factor for early recurrence in breast cancer. *Cancer Res.*, **37**, 4669.

Lowry, O. H., Rosebrough, N. R., Farr, A. L. & Randall, R. J. (1951) Protein measurement with the Folin phenol reagent. *J. Biol. Chem.*, **193**, 265.

McGuire, W. L., Carbone, P. P. & Vollmer, E. P. (1975) (Editors) *Estrogen Receptors in Human Breast Cancer*. New York: Raven Press.

Rosen, P. P., Menendez-Botet, C. J., Nisselbaum, J. S. & 4 others (1975) Pathological review of breast lesions analysed for estrogen receptor protein. *Cancer Res.*, **35**, 3187.

Wolff, B. (1966) Histological grading in carcinoma of breast. *Br. J. Cancer*, **20**, 36.